# Effects of *Ficus pandurata* Hance var. *angustifolia* Cheng Flavonoids on Intestinal Barrier and Cognitive Function by Regulating Intestinal Microbiota

**DOI:** 10.3390/foods12081682

**Published:** 2023-04-18

**Authors:** Yuting Zhang, Junjie Pan, Yanan Liu, Xin Zhang, Kejun Cheng

**Affiliations:** 1 Department of Food Science and Engineering, Ningbo University, Ningbo 315211, China; 2 Chemical Biology Center, Lishui Institute of Agriculture and Forestry Sciences, Lishui 323000, China

**Keywords:** *Ficus pandurata* Hance var. *angustifolia* Cheng flavonoids, intestinal barrier, intestinal microbiota, cognitive function

## Abstract

More and more evidence has supported the interaction between circadian rhythms and intestinal microbes, which provides new insights into how dietary nutrition can improve host health. Our research showed that *Ficus pandurata* Hance var. *angustifolia* Cheng flavonoids (FCF) ameliorated the pathological damage of colon and abnormal intestinal microflora structure in mice with circadian clock disorder and improved their exploration and memory behaviors. Mechanism studies have shown that FCF is involved in regulating metabolic pathways and related metabolites, regulating the expression of related tight junction proteins in the colon and the levels of Aβ and inflammatory factors in the hippocampus. Further analysis found that these metabolites showed a certain correlation with intestinal flora and played a certain role in alleviating intestinal physiological damage and cognitive decline.

## 1. Introduction

Circadian rhythm means that organisms evolve endogenous rhythm oscillations of about 24 h to adapt to the alternating changes of day and night caused by the Earth’s rotation [1]. The primary function of circadian rhythms is to anticipate daily changes in the external environment, thereby maintaining homeostatic balance and ensuring an adaptive physiological response to fluctuating environments. With the accelerating pace of social life and the increasing pressure of competition, many occupations implement the shift system, and more and more people are engaged in shift work [2]. The most direct and early effect of circadian rhythm disorder is to cause corresponding psychological and physiological damage to the human body, and long-term circadian rhythm disorder will increase the risk of cancer, cardiovascular disease, metabolic syndrome and so on [3]. Meanwhile, circadian disruption caused by environmental and genetic factors will lead to increased intestinal permeability and intestinal flora disorders [4]. Intestinal microflora and intestinal mucosa and intestinal epithelial cells form a complex and stable dynamic relationship, promote intestinal mucosal and protect the host from pathogens, which is of great significance to body health [5]. Evidence of biological clock gene expression in brain cells other than the suprachiasmatic nucleus (SCN) indicates that biological clocks play a fundamental role in cognitive performance, learning and emotion. As an important brain area carrying human cognitive function, the hippocampus participates in the acquisition and maintenance of memory [6]. The process of hippocampal biological function impairment caused by circadian rhythm disorder is closely related to hippocampal neurogenesis, synaptic plasticity-related protein synthesis and neuroinflammation [7].

The intestinal mechanical barrier is composed of mucosal epithelial cells and intercellular junctions, in which tight junctions are composed of connective proteins located in intercellular channels of intestinal cells [8]. In addition, the integrity of the intestinal barrier is the key to maintaining the homeostasis of the digestive tract and resisting the invasion of pathogenic microorganisms and microbial toxins [9]. The number of intestinal microorganisms is huge, and the number of human intestinal flora is as high as 10^13^–10^14^, which is tens of times that of human cells [10]. In recent years, several studies have shown that changes in intestinal microbiota will affect the function of neurons, thus affecting the behavior of the host [11]. Researchers compared pathogen-free and sterile mice with wild-type mice and found that the former had many cognitive deficits, including impaired spatial and working memory impairment [12]. Furthermore, intestinal microflora can release a large amount of amyloid, lipopolysaccharide (LPS) and other microbial metabolites into the surrounding tissues. The absorption of these molecules is related to the signal pathways related to the production of pro-inflammatory cytokines, some of which are related to Alzheimer’s disease (AD). In addition, the increase of bacterial amyloid protein may lead to the progress of Aβ accumulation in the brain [13].

Flavonoids have a broad spectrum of pharmacological activities and a wide range of biological effects, such as antioxidant effects, which can prevent cardiovascular diseases and cancer [14]. They can also improve inflammatory bowel disease by inhibiting harmful bacteria and increasing the abundance of *Bifidobacteria* and other probiotics [15]. Currently, the regulatory mechanisms of flavonoid natural products on intestinal immunity include maintaining the integrity of intestinal mucosa, activating signal pathways related to the intestinal mucosal barrier in intestinal epithelial cells and preventing the invasion of pathogens [16]. Noteworthy, flavonoids extracted from many fruits and vegetables have been proven to improve memory and learning. These compounds directly act on receptors, kinases and transcription factors in brain tissues related to memory, as well as inhibit the production of inflammatory factors in glial cells in the state of inflammation activation, thereby improving cognitive function [17]. *Ficus pandurata* Hance var. *angustifolia* Cheng is a plant of the genus Ficus of Moraceae, which is a commonly used herbal medicine in southwest Zhejiang Province of China, and it is also one of the most commonly herbal medicines of the nationality. According to domestic and foreign research, as a banyan plant, the content of flavonoids in *Ficus pandurata* Hance var. *angustifolia* Cheng is high [18]. Flavonoids extracted from Ficus of Moraceae have been reported to exert anti-inflammatory, antibacterial, lipid-regulate and strong immune activities, which is a functional component worthy of further study [19]. Some recent studies have demonstrated that flavonoids can prevent and even treat circadian rhythm-related diseases and act as circadian rhythm regulators [20].

Therefore, our research aimed to investigate the regulatory effects of flavonoids of *Ficus pandurata* Hance var. *angustifolia* Cheng (FCF) on intestinal flora in mice with circadian disturbance, focusing on the metabolic level-related regulation of intestinal barrier and brain inflammatory proteins. Based on a new perspective of intestinal flora and metabolic level, the potential pathogenesis of intestinal damage and cognitive decline caused by circadian rhythm disturbance was explored, providing a reliable basis for theoretical research on the protection of intestinal tract and cognitive ability by flavonoids in Ficus through intestinal flora.

## 2. Materials and Methods

### 2.1. Materials and Reagents

*Ficus pandurata* Hance var. *angustifolia* Cheng was collected from Lishui City, Zhejiang Province, China. Polyamide resin was produced in Ocean Chemical Co., Ltd. (Qingdao, China). The experimental mice came from Beijing Vital River Laboratory Animal Technology Co., Ltd. (Beijing, China). Ningbo Experimental Animal Center provided the ordinary animal feed. The detection ELISA kits of IL-1β, IL-10 and IL-6 were purchased from MultiSciences Biotechnology Co., Ltd., Hangzhou, China. ZO-1, occludin and Aβ kits were manufactured by Elabscience Biotechnology Co., Ltd. (Wuhan, China). Protein antibodies of ZO-1, occludin and Aβ were produced in Servicebio Technology Co., Ltd. (Wuhan, China). The other chemical reagents used in the experiment are all analytical pure grade.

### 2.2. Preparation of Flavonoids from Ficus pandurata Hance var. angustifolia Cheng

We took the appropriate amount of *Ficus pandurata* Hance var. *angustifolia* Cheng powder and added the corresponding volume of 70% ethanol at 1:40. Ultrasonic-assisted extraction was performed for 50 min, followed by water bath extraction for 2 h. The extract was centrifuged to obtain the supernatant and the filter residue was extracted again according to the above method. Finally, we combined the twice-clear supernatant. The obtained supernatant was adsorbed by polyamide resin and desorbed with 80% ethanol solution. Then, classification and purification were carried out by polyamide column. After passing the column, the filtrate was evaporated by rotation and freeze-dried to obtain an FCF sample, which was stored at −20 °C for later use. The chemical contents of FCF were determined according to the reported method [21,22].

### 2.3. Animal Experiment Design

SPF C57BL/6J male mice (6–8 weeks of age) were used as laboratory animals in the Experimental Animal Center of Ningbo University (permission no. SYXK [Zhejiang] 2013–0191). They were fed in the sterile barrier facilities at a temperature of 22–24 °C and humidity of 60 ± 5%. After 1 week of initial accommodation, the mice were randomized into 3 groups (10 in each group): 12 h light-dark cycle control group (CT group), constant darkness group (CD group) and constant darkness with intragastric administration of 0.1% (*w*/*w*) FCF group (FCF group). Body weight, food and water intake were recorded weekly. In addition, stool samples were collected and stored at −80 °C, at week 0 and week 4, respectively. Four weeks later, behavioral experiments were carried out.

### 2.4. Behavioral Assessment

After darkness adaptation, mice were placed in the central zone of the Y-maze device and explored freely in the Y maze for 8 min. The sequence and the total number of arms were recorded, and the correct spontaneous alternation rate was calculated. The open-field test was used to test the free exploration activity and sensorimotor ability of mice. The size of the open field is 40 cm × 40 cm × 40 cm, which was divided into two parts: the central area of 13.5 cm × 13.5 cm and the surrounding area. The infrared camera was set on the top to record the activities of the mice. Detailed operation methods are presented in the Supporting Information. 

### 2.5. Experimental Tissue Sampling

After the animal experiment, the mice were euthanized. After the eyeballs of the mice were removed, the cecal melt was collected. Then, the brain and colon tissues were stripped and fixed in 4% paraformaldehyde. Subsequently, the hippocampal tissues were separated on ice and placed in cryo-storage tubes and then stored all the above tissues in a −80 °C refrigerator.

### 2.6. Enzyme-Linked Immunosorbent Assay (ELISA)

Colon tissue and hippocampal tissue were collected and homogenized on ice, and the supernatant was taken after centrifugation according to the instructions of the ELISA kit. The levels of tight junction proteins ZO-1 and occludin in the colon, Aβ and inflammatory factors were determined. The extraction buffer was extracted with 0.05 mol/L carbonate solution (0.159 g sodium carbonate, 0.294 g sodium bicarbonate, diluted to 100 mL with distilled water).

### 2.7. Histological Examination

We took the colon tissues and hippocampus tissues of mice and embedded them and make slices. The sections were placed into some kinds of BioDewax and Clear Solution according to the operating instructions in Supporting Information. Finally, the microscopic examination was performed, and images were collected and analyzed.

### 2.8. Detection of Occludin and ZO-1 Expression in Intestinal Tissue by Immunohistochemical Method

The colon tissue was embedded in paraffin and sectioned. After the antigen was repaired, endogenous peroxidase was blocked and the serum was blocked for 30 min. The primary antibody prepared in a certain proportion with PBS was dropped and incubated overnight at 4 °C. The primary antibody was prepared with 0.4% PBS at a 1:200 ratio, and the secondary antibody was prepared with 0.4% PBS at a 1:500 ratio. After incubation, rinse and shake the slices, we added the covering tissue of the second antibody (HRP marker) of the corresponding species, incubated them at room temperature for 50 min and then DAB was used for color rendering. The nucleus was re-stained with hematoxylin and then returned to blue with hematoxylin solution and washed with running water. Finally, the seal film was dehydrated and observed under an optical microscope.

### 2.9. Immunofluorescence Detection Test

Firstly, the paraffin hippocampus tissue sections were dewaxed in water to repair the antigens. Then, the sections were slightly shaken dry, and a histochemical pen was used to draw a circle around the tissues. The sealing solution was removed after sealing for 30 min, adding the first antibody of PBS in a certain proportion to the slices and putting the slices flat in a wet box at 4 °C to incubate overnight. In addition, PBS was added to the sections with a certain proportion of primary antibody, and the sections were placed horizontally in a wet box for incubation at 4 °C overnight. Among them, the primary antibody was prepared with 0.01 mol/L PBS at a 1:200 ratio, and the secondary antibody was prepared with 0.01 mol/L PBS at a 1:100 ratio. After incubation, the slides were washed and dried in PBS, then the second antibody covering tissue of the corresponding species was added to the circle and incubated in the dark for 50 min at room temperature. After that, the nucleus was re-stained with 4-diaminophenyl indole (DAPI), followed by an autofluorescence quenchant. Finally, the tablets were sealed with an anti-fluorescence quenching agent, the slices were observed under a fluorescence microscope and the images were collected.

### 2.10. Analysis of Intestinal Flora

The methods of DNA extraction and high-throughput sequencing referred to the methods we reported earlier, and some programs have been modified appropriately [23]. Detailed operation methods are presented in the Supporting Information. The species composition of different taxa and the structural composition of important floras at different taxonomic levels were analyzed.

### 2.11. Metabolite Extraction and LC−MS Analysis

Cecal contents were collected for metabonomic analysis. Firstly, the samples were thawed, and metabolites were extracted from the samples by pre-cooled 50% methanol buffer. Then, the mixture of metabolites was vortexed for 1 min, then incubated at room temperature for 10 min and stored overnight at 20 °C. In addition, the sample was centrifuged at 4000× *g* for 20 min. Subsequently, the supernatant was transferred to a 96-well plate [24]. Finally, the sample is kept at −80 °C for LC-MS analysis. Detailed operation methods are presented in the Supporting Information. MetaX was used to further pre-process the peak intensity data, and the preprocessed data were used for principal component analysis to detect anomalies and batch-processing effects.

### 2.12. Statistical Analysis

At least 3 replicates were performed for each set of data, and the data obtained were analyzed using SPSS (SPSS Inc., Chicago, IL, USA, v 17.0.0). Significant differences between groups were performed by one-way ANOVA. The differences among the groups were evaluated using Tukey’s test, *p* < 0.05 was considered statistically significant.

## 3. Results

### 3.1. The Contents of FCF in the Extract

The composition analysis of FCF was shown in Table 1 and the chromatogram was shown in Figure S1. It could be seen that the content of rutin and vitexin in FCF extract were 169.77 ± 13.27 and 143.52 ± 14.81 mg/g, which were the dominant flavonoid in the extract. In addition, the content of apigenin in FCF extract was 119.98 ± 11.51 mg/g. The contents of epicatechin and luteolin were lower at 38.69 ± 1.63 and 23.41 ± 0.85 mg/g.

### 3.2. Effect of FCF on Body Weight and Caloric Intake in Mice with Circadian Rhythm Disorder

**Figure 1:**
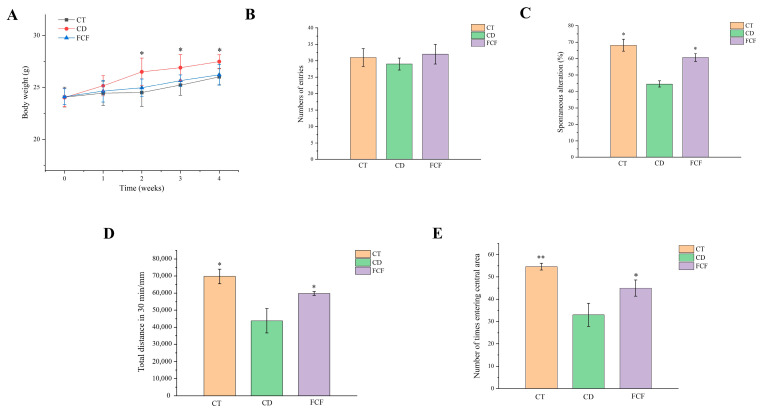
(**A**) Effect of FCF on the body weight of circadian rhythm disorder mice. * *p* < 0.05, compared with the CD group. The effect of FCF on the total number of arm entries (**B**) and the correct rate of spontaneous alternation (**C**) in mice with circadian rhythm disorder. The effect of FCF on the total 30 min exercise distance (**D**) and the times of entering the central area (**E**) in mice with circadian rhythm disorder. * *p* < 0.05, ** *p* < 0.01, compared with the CD group.

In Figure 1A, it can be seen that after 2 weeks of feeding mice, the average body weight of the CD group was higher than that of the CT group (*p* < 0.05), and there was no obvious difference in body weight between FCF group and CT group (*p* > 0.05). From weeks 2 to 4, compared with mice in the CD group, the body weight of the FCF group grew more slowly (*p* < 0.05). However, there was no significant difference in the intake of water and food among the three groups (*p* > 0.05), indicating the direct effect of FCF on weight loss (Figure S2).

### 3.3. Effects of FCF on Cognitive Activity in Mice with Circadian Disturbance

In the Y-maze test, as shown in Figure 1B, there was no significant difference in the total number of arm entries among the three groups (*p* < 0.05). The accuracy of spontaneous arm alternation was 44.6 ± 1.9% in the CD group, which was significantly lower than that in the CT group (68.2 ± 3.6%) (*p* < 0.05) (Figure 1C). In addition, the correct rate of spontaneous alternation in FCF group was increased to 58.5 ± 2.4%. The results showed that compared with the CT group and FCF group, the mice in CD group had a weaker desire to explore and short-term working memory ability. These results suggested that FCF can alleviate the short-term working memory impairment of mice with circadian rhythm disorder, but has no obvious effect on their exercise ability. An open-field experiment is a method to evaluate the independent inquiry behavior and tension of experimental animals in a novel environment. In Figure 1D, compared with the CT group, the total 30 min exercise distance of CD mice was reduced (*p* < 0.05), and FCF intervention effectively increased the total distance of mice in the CD group (*p* < 0.05). The number of mice in the CD entering the central area was less than that in the CT group (*p* < 0.05). Although the frequency of mice entering the central area of FCF group was lower than that of CT group, it was significantly higher than that in the CD group (Figure 1E).

### 3.4. FCF Improved Intestinal Barrier Function in CD Mice

#### 3.4.1. Intestinal Histopathological Changes in Each Group

**Figure 2:**
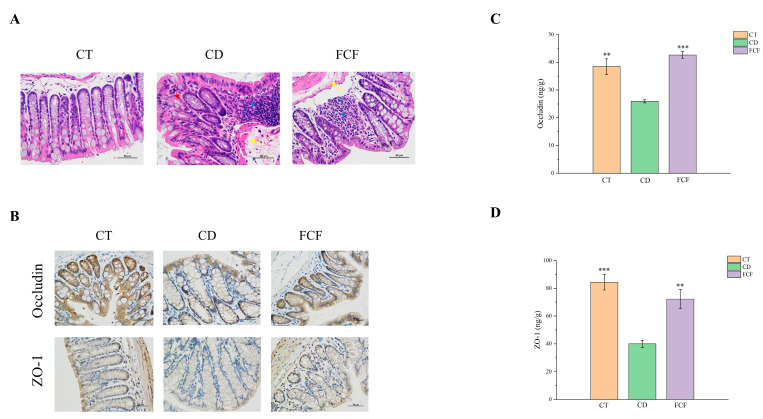
(**A**) Effect of FCF supplementation on intestinal histomorphology in mice with circadian rhythm disruption (HE, ×400). (**B**) Immunohistochemistry of occludin and ZO-1 of the three groups (Immunohistochemistry, ×400). Effects of FCF on the expression of tight junction proteins occludin (**C**) and ZO-1 (**D**) in the colon of mice with circadian rhythm disorder. ** *p* < 0.01, *** *p* < 0.001, compared with the CD group.

It can be observed from the HE staining results (Figure 2A) that the intestinal submucosa of the CT group was slightly edematous, but no obvious histopathological changes were found. In the CD group, eosinophilic degeneration could be seen in mucosal epithelial cells, goblet cells were reduced in number and glandular structure was slightly atrophic. In group CD, eosinophilic degeneration of mucosal epithelial cells was observed, glandular structure was slightly atrophied and the number of goblet cells was reduced (red arrow). There was more lymphocyte infiltration in lamina propria (blue arrow), slight hyperplasia of fibrous tissue in mucosal layer and mild submucosal edema with vascular dilatation (yellow arrow). As for the FCF group, there was mild edema in the mucosa and submucosa of the colon (yellow arrow), with a small amount of inflammatory infiltration (blue arrow). The intestinal tissue injury score in the CD group was significantly higher than that in the CT group (*p* < 0.05). Compared with the CD group, the intestinal tissue damage of mice in the FCF group was reduced, as shown in Figure S3.

#### 3.4.2. The Expression of Occludin and ZO-1 in Intestinal Tissues of Mice in Each Group

The immunohistochemical analysis illustrated that tight junction protein was positively expressed in the intestinal and glandular epithelial cells of the three groups, as well as in the proximal cytoplasmic region of the membrane, with more brownish-yellow staining (Figure 2B). Compared with CT and FCF group, the expression of occludin in intestinal and glandular epithelial cells in the CD group decreased. ZO-1 was positively expressed in the cytoplasmic area of intestinal and glandular epithelial cells in the CT group, with more brown-yellow staining. In addition, compared with the FCF group, there was increased inflammation and decreased ZO-1 protein expression in the CD group. To be more specific, ELISA analysis showed the expression level of occludin (Figure 2C) and ZO-1 (Figure 2D) in the CD group was observably lower than in the CT group (*p* < 0.01). In comparison with the CD group, the expression of occludin and ZO-1 in the FCF group was significantly increased (*p* < 0.01).

### 3.5. Effects of FCF on the Diversity and Structure of Intestinal Flora in Mice with Circadian Rhythm Disturbance

**Figure 3:**
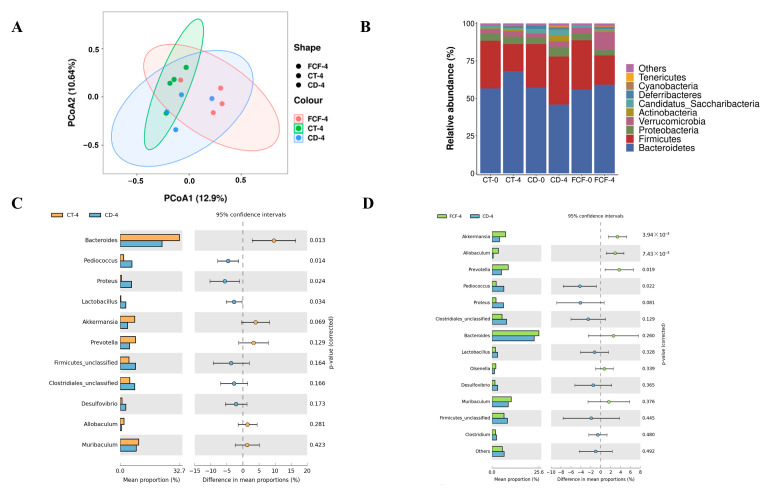
(**A**) Principal component analysis of fecal microbiota of the three groups. (**B**) The relative abundance of intestinal flora at the phylum in each group (Weeks 0 and 4). The comparison of relative abundance of the intestinal microbiota at genus in the 4th week between CD and CT group (**C**), CD and FCF group (**D**).

Alpha diversity is generally used to indicate the richness and evenness of species in a community ecosystem. The results of the alpha diversity index were shown in Table 2. After 4 weeks, the Chao1 index in the CD group was significantly lower than that of the other two groups (*p* < 0.05). At week 4, Shannon and Simpson’s indices indicated that fecal flora diversity in the FCF group was significantly higher than that in the CD group (*p* < 0.05), and Shannon’s index in the CD group decreased after 4 weeks. The results suggested that continuous darkness reduced the species richness and diversity of the intestinal microflora of mice, while FCF supplementation increased the species diversity of the intestinal microflora. Based on PCoA analysis results (Figure 3A), different colors in the figure represent different groups. The closer the distance between samples is, the more similar the composition and structure of microorganisms are among the samples. It can be seen that the community structure similarity of the CD group was low, and FCF feeding treatment effectively reversed the differences in microbial composition caused by CD.

The results of 16S rDNA sequencing showed that long-term circadian disturbance caused changes in the species composition and community structure of intestinal microflora in mice. At the phylum level, in Figure 3B, *Bacteroidetes*, *Firmicutes* and *Proteobacteria* were the dominant flora in the intestinal tract of mice. After 4 weeks, the relative abundance of *Bacteroidetes* in the CD group was significantly lower than that in the CT group and FCF group (*p* < 0.05), and the relative abundance of *Firmicutes* changed on the contrary. After 4 weeks of FCF treatment, the corresponding ratio of *Firmicutes/Bacteroidetes* (F/B) decreased from 0.59 to 0.33, showing that FCF had a great inhibitory effect on the growth of *Firmicutes*. Compared with the disorder group, the ratio of F/B in the control group also decreased. What is more, we implemented statistical analysis on the relative abundance of microflora in the samples at the genus level (Figure 3C,D). Under 4 weeks of dark treatment, the relative abundance of *Bacteroides* decreased significantly (*p* < 0.05), and the relative abundance of *Bacteroides* increased after feeding FCF. Moreover, FCF treatment could significantly reverse the decline in the relative abundance of *Akkermansia*, *Allobaculum* and *Prevotella* caused by continuous darkness (*p* < 0.05). In addition, compared with the CD group, the relative abundance of *Pediococcus* in the FCF group decreased (*p* < 0.05). Therefore, FCF intervention can effectively alleviate the abnormal relative abundance of the predominant intestinal microbiota.

### 3.6. Influences of FCF on Intestinal Metabolites in Mice with Rhythm Disturbance

**Figure 4:**
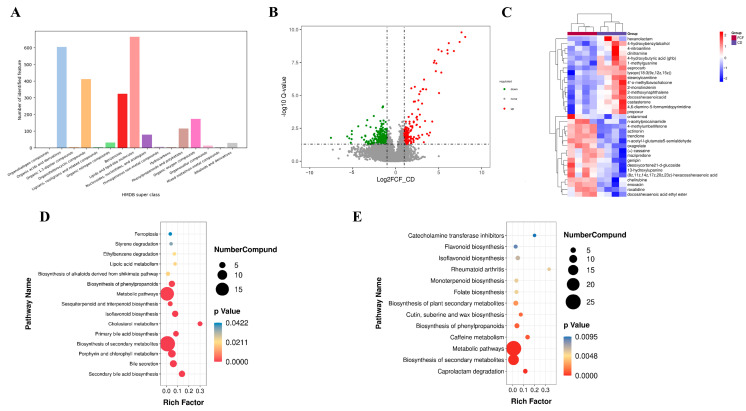
(**A**) HMDB super class identification classification and annotation diagram of metabolites. (**B**) Volcano plots of the different metabolites in the FCF and the CD groups. (**C**) Heatmap of the different metabolites between the FCF group and the CD group. (**D**) KEGG enrichment pathways of different metabolites between the CD and CT groups. (**E**) KEGG enrichment pathways of regulated differential metabolites between the CD group and FCF group.

According to superclass analysis of the human metabolome database (HMDB), the most abundant metabolites identified by us are mainly lipids and functions-like molecules, organic acid and derivatives and organoheterocyclic compounds (Figure 4A). The close grouping of QC samples in the PCA chart further confirmed the high repeatability and stability of the whole testing process (Figure S4), and the metabolites of the three groups could be divided, which manifested the good data quality. In addition, the results of mass spectrometry showed that different metabolites were obtained under the conditions of PIM and NIM (Figure 4B). After the screening, we obtained 33 secondary metabolites (16 down-regulated metabolites and 17 up-regulated metabolites) with significant differences between FCF and CD group (Figure 4C). It can be observed that the most important feature of FCF intervention is the up-regulation of metabolites beneficial to neuroprotection and anti-inflammatory effects, including actinonin, 4-methylumbelliferone (carbohydrate polyketones), genipin (lipids and lipid-like molecule) docosahexaenoic acid ethyl ester (DHA-ee) (lipids and lipid-like molecule) and enoxacin. In contrast, after FCF supplementation, some pro-inflammatory, neuropathic metabolites were down-regulated, including some lipids and lipid molecules like lysopc (18:3 (9z,12z,15z)), stearoylcarnitine, stearoylcarnitine, stearoylcarnitine, 2-monolinolenin and 2-methoxynaphthalene (benzenoids), 4,6-diamino-5-formamidopyrimidine, 4-hydroxybutyric acid. It was found that after treatment under constant darkness, metabolite enrichment pathways with significantly decreased metabolic levels mainly include cholesterol metabolism, secondary bile acid biosynthesis, isoflavonoid biosynthesis, etc. (Figure 4D). After FCF intervention in mice, the identified enrichment pathways of differential metabolites were catecholamine transferase inhibitors, isoflavone biosynthesis and caffeine metabolism under the condition of continuous darkness (Figure 4E).

### 3.7. Correlation between Intestinal Microflora and Intestinal Metabolites

**Figure 5:**
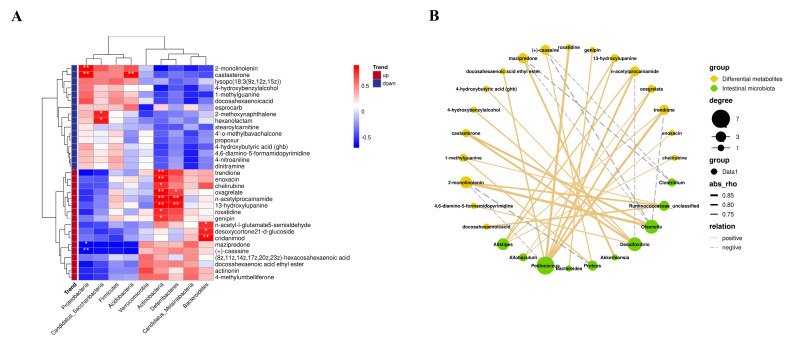
(**A**) The correlation between intestinal microbiota at phylum and differential metabolites between the CD group and FCF group * *p* < 0.05, ** *p* < 0.01, compared with the CD group. (**B**) Network diagram of intestinal differential metabolites and microbiota at the genus levels.

As shown in Figure 5A, *Bacteroidetes*, *Proteobacteria*, *Acidobacteria*, *Proteobacteria* and *Deferribacteres* were associated with significant differences in intestinal metabolites between the CD and FCF groups. The metabolites that were significantly positively correlated with *Bacteroidetes* were DHA-ee, n-acetyl-L-glutamate and 5-semialdehyde (*p* < 0.05). The metabolites that showed a significant positive correlation with *Deferribacteres* were oxagrelate and n-acetylprocainamide (*p* < 0.01). Interestingly, the above metabolites are also positively correlated with *Acidobacteria* (*p* < 0.01). Figure 5B showed the correlation network diagram between differential metabolites and gut microflora in the CD group and FCF group. Metabolites positively correlated with *Pediococcus* include roxatidine, n-acetylprocainamide and oxagrelate. Genipin was significantly positively correlated with *Bacteroides*. *Akkermansia* was positively correlated with mazipredone, and *Allobaculum* was positively correlated with oxagrelate and enoxacin. Our data reflected that there was a good correlation between intestinal flora and intestinal metabolites. Combined with the diagram (intestinal microbiota at phylum level and genus level), FCF helped gut flora to repair the intestinal environment and promote the production of beneficial metabolites.

### 3.8. Physiological Mechanism of FCF Improving Cognitive Function in Mice with Circadian Disturbance

#### 3.8.1. Morphological Comparison of Hippocampal Neurons in Each Group

**Figure 6:**
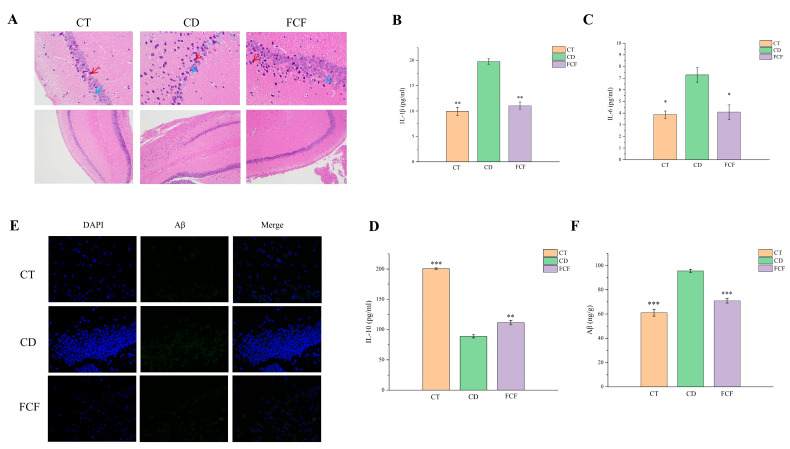
(**A**) Effects of FCF on hippocampal histomorphology in mice with circadian disturbance (HE, ×200 and ×400). Effects of FCF on the contents of proinflammatory factor IL-1β (**B**), IL-6 (**C**) and anti-inflammatory IL-10 (**D**) in the hippocampus of mice with circadian rhythm disturbance. (**E**) Results of Aβ fluorescence staining in the hippocampus of each group (×400). (**F**) Effect of FCF on hippocampal Aβ level in mice with circadian disturbance. * *p* < 0.05, ** *p* < 0.01, *** *p* < 0.001, compared with the CD group.

HE staining (Figure 6A) showed that hippocampal neurons of mice in the CT group were arranged neatly, with some nuclei being shriveled (red arrows) and occasional necrosis (blue arrows). The number of cells in CD group decreased and their arrangement was loose and disordered. Numerous nuclei were shrunken (red arrows), the nucleoli were not clear and necrosis was noted (blue arrows). Neurons in the FCF group were disordered, with some nuclei shrunken and hyperchromatic (red arrows) and displaying necrosis (blue arrows).

#### 3.8.2. Expression of Inflammatory Factors in the Hippocampus of Each Group

As shown in Figure 6B, ELISA results revealed that IL-1β expression in the hippocampus of mice in the CD group was significantly higher than that in the CT group (*p* < 0.01), compared with the CD group, IL-1β level in the FCF group was markedly decreased. In addition, the IL-6 expression level in the CD group was higher than that in the CT group (*p* < 0.05), IL-6 level was significantly decreased after FCF supplementation (*p* < 0.05) (Figure 6C). The expression of IL-10 in the hippocampus of CD group was significantly lower than that of CT group (*p* < 0.001), and the levels of IL-10 was significantly higher in the FCF group than that of CD group (*p* < 0.01) (Figure 6D).

#### 3.8.3. Expression of Aβ in the Hippocampus of Each Group

Immunofluorescence staining was used to detect the contents of Aβ protein in the brain of mice. It was found that there were few Aβ plaques in the brain of mice in the CT control group and a high level of Aβ plaques in the brain of mice in the CD model. However, FCF-treated CD model mice significantly reduced the content of Aβ in the brain and the deposition of Aβ plaque in the hippocampus (Figure 6E). We detected the expression level of Aβ in hippocampal tissues of each group by ELISA (Figure 6F). The results showed that the content of Aβ in CT group was 61.11 ± 2.80 ng/g, and the expression intersection of Aβ in CD group was significantly higher than that in FCF group, reaching 95.67 ± 1.40 ng/g (*p* < 0.001).

## 4. Discussion

It has long been reported that SCN can coordinate metabolic processes and energy balance through the endocrine and autonomic nervous systems [25]. Evidence shows that sleep deprivation and circadian rhythm disorder are closely related to metabolic disorders, which will lead to obesity [26]. Our experimental results are consistent with the above studies, suggesting that a continuous dark environment can cause weight gain in mice, and this result may be related to metabolic disorder and energy imbalance. Whereas our study found that FCF supplementation was effective in slowing weight gain caused by a circadian disruption in mice, dietary flavonoid supplementation has been shown to reduce weight in high-fat diets (HFD) [14]. Circadian rhythm also affects the body’s ability to learn and remember, and the mouse model with circadian rhythm gene disruption showed poor performance in various learning and behavior tasks [2]. After 4 weeks of FCF supplementation, short-term memory impairment and decreased spatial exploration were improved in mice with circadian disruption. Flavonoids have been conducted to modulate activity-dependent synaptic protein synthesis and improve the morphology of nerve cells and nerve fibers, thereby altering the neurodegeneration process and the acquisition and storage of memories [27].

The change in intestinal microbiota is characterized by the increase of pro-inflammatory bacteria, the decrease of bacteria that can produce anti-inflammatory short-chain fatty acids and the imbalance of the F/B ratio [28]. In this study, a significant increase in *Proteobacteria* and *Actinobacteria* was also observed in CD mice. In addition, 4 weeks of FCF intervention significantly increased the relative abundance of *Bacteroidetes* and *Verrucomicrobia* in mice with circadian rhythm disorder. The microflora are the main producers of short-chain fatty acids in the human intestines, mainly in the form of propionate and butyrate, which play an important role in maintaining intestinal homeostasis [29]. Acetate and propionate are both effective anti-inflammatory mediators because they inhibit neutrophils and macrophages from releasing pro-inflammatory cytokines. Propionate can induce apoptosis of human colon cancer cells, and butyrate increases intestinal tight junction protein expression to reduce underlying intestinal permeability, which, in turn, reduces inflammation and endotoxemia associated with intestinal leakage [30]. *Bacteroides* is involved in many important metabolic activities in the human colon, including the fermentation of carbohydrates, the utilization of nitrogen-containing substances and the biotransformation of bile acids and other steroids [31]. The abundance of *Actinobacteria* was also found to be associated with better reaction time and attention, while an increase in *Prevotella* led to increased reaction time and poor attention. Researchers conducted an exploratory study and found that intestinal flora was positively correlated with sleep quality and cognitive activity. The higher proportion of *Verrucomicrobia* and *Lentisphaerae* in intestinal flora was related to better cognitive flexibility in healthy elderly people [32]. We also observed that FCF treatment significantly reversed the decline in the relative abundance of *Akkermansia*, *Allobaculum* and *Prevotella* induced by continuous darkness. In addition, the relative abundance of *Pediococcus* in the FCF group was less than that in the CD group. *Allobaculum* was identified as an active glucose assimilate. In addition, C^13^-labeled glucose was fermented by *Allobaculum*, and it was found that the main metabolites were acetate, propionate and lactate [33]. *Akkermansia* belongs to the phylum *Verrucomicrobia*, which is one of the most widely studied probiotics and may be suitable for the treatment of the metabolic syndrome. *Akkermansia* produces acetate, the propionate and ethanol from mucin fermentation to regulate host biological functions, including host immune response and lipid metabolism [34]. Many studies have found that patients with inflammatory bowel disease (IBD) have similar intestinal microbial change patterns, including the decline of microbial diversity. The severity of IBD is closely related to the relative increase of *Proteobacteria* and the relative decrease of *Clostridium* and *Akkermansia* [35]. It is worth noting that *Ackermania* has played the role of “probiotics” in the occurrence and development of neurodegenerative diseases in recent years. A mechanism study explored the effects of *Akkermansia* administration in a mouse model of AD fed an HFD. In addition to its anti-obesity effect, *Akkermansia* inoculation significantly reduced Aβ in the brain and improved performance on cognitive tests regardless of the dietary pattern [36].

The function of the intestinal mucosal mechanical barrier mainly depends on the integrity of epithelial cells. In the case of intact epithelium, the permeability of the intestinal epithelial barrier is mainly determined by the tight junction between cells [37]. As important structural proteins of tight junctions, the abnormal expression of ZO-1 and occludin may hinder the formation of tight junction proteins between cells, destroy the intestinal mechanical barrier and cause pathogens and bacterial toxins to flow into the blood [38]. The circadian rhythm disorder led to submucosal edema in the colon tissue of mice in the present study, which reduced the expression of tight junction protein and then damaged the intestinal mucosal mechanical barrier. After FCF intervention, the expression of ZO-1 and occludin in the intestinal epithelial cells was up-regulated, and the shape of the colon was restored to a certain extent. Intestinal tight junction proteins are sensitive to changes in the gut flora, and the increase of intestinal permeability will cause harmful substances such as bacterial endotoxins and heterologous antigens to enter the internal environment through the mucosal barrier, causing intestinal inflammation eventually [39]. Previous studies demonstrated that luteolin can increase transepithelial resistance, reduce Lucifer yellow flux and up-regulate the expression of tight junction proteins such as ZO-1, occludin and claudin-1, thereby reducing ethanol-induced intestinal barrier damage. Mechanism research reveals that luteolin can inhibit phosphorylation of myosin light chain 2, the activity of myosin light chain kinase and the nuclear translocation of NF-κB [40]. Puerarin belongs to the isoflavone compound extracted from Chinese herbal medicine, which is involved in up-regulating ZO-1 protein expression in rats with intestinal barrier dysfunction [41].

Inflammatory responses are initiated and promoted by inflammatory factors. We investigated the changes of IL-6, IL-1β and IL-10 in the hippocampus of mice with circadian rhythm disorder after FCF intervention. According to the results of our experiment, we speculated that circadian rhythm disorder could stimulate the inflammatory response in the hippocampus, and FCF, as a flavonoid, reduced inflammation. Hippocampal inflammation is involved in the development of anxiety and cognitive dysfunction in mice [42]. In a study, quercetin played a role in chronic unpredictable stress, which induced oxidative stress and neuroinflammation in mice with hippocampal behavioral dysfunction [43]. The results demonstrated that oral administration of quercetin at 30 mg/kg reduced the level of pro-inflammatory cytokine (IL-6, TNF- α, IL-1β and COX-2) and prevented nerve injury. Moreover, pretreatment of mouse primary microglia and BV2 microglia with luteolin can inhibit LPS stimulating IL-6 production at mRNA and protein levels [44]. EMSA (electrophoretic migration assay) showed that this flavonoid strongly inhibited the binding of activator protein 1 (AP-1) on the IL-6 promoter, and LPS stimulated microglia to produce IL-6 and inhibit jun N-terminal kinases (JNK) phosphorylation [45].

Aβ can induce neuronal apoptosis, peroxidation damage, brain inflammation and other neurotoxic effects. Previous studies have shown that the abundance of Aβ fluctuates with circadian rhythm [46]. Under physiological conditions, the concentration of Aβ is low and it is a kind of soluble substance. A lot of them were produced under pathological conditions and changed from soluble form to insoluble β-fold structure, gradually accumulating to form the core of senile plaque [47]. Moreover, studies have also shown that Aβ deposition in neurons is closely related to the changes in synaptic function and spatial learning and memory of neurons [48]. In this experiment, continuous darkness was used to cause circadian rhythm disorder in mice, and it was found that it could increase the content of Aβ in the hippocampus of mice, accompanied by spatial memory and exploration disturbance. It is suggested that this non-invasive animal model can show the characteristic pathological changes of cognitive impairment-Aβ deposition. After supplementation of FCF, the level of Aβ in the CD group tended to be that of the normal control group. Interestingly, flavonoids can exert neuroprotective effects by activating the non-amyloid pathway and inhibiting the amyloid pathway, thus inhibiting the activity of BACE-1 and reducing the formation of Aβ [48]. Researchers showed that baicalein can inhibit the formation and aggregation of Aβ plaque in the brain of AD model rats, depolymerize the formed Aβ plaque and significantly reduce the toxic effect of Aβ on cells [49].

Our experiment found that circadian rhythm disorder resulted in significant abnormalities of cholesterol metabolism and secondary bile acid biosynthesis. The content of cholesterol in mammalian serum has the characteristic of circadian rhythm, and the steroids produced by cholesterol conversion also have a similar circadian rhythm [26]. The above results suggest that cholesterol metabolism is regulated by the biological clock system. Disruption of circadian rhythms can alter the metabolism of bile acids and cause intestinal inflammation, resulting in cholestatic liver damage and intestinal micro imbalances. Poor secretion of bile acid directly leads to abnormal lipid metabolism, excessive growth of intestinal harmful bacteria and serious metabolic disorders [5]. Differential metabolites were significantly accumulated in metabolic pathways of catecholamine transferase inhibitors and isoflavonoid biosynthesis. Catecholamine transferase inhibitors can inhibit the metabolic degradation of catecholamines [50]. In recent years, a variety of experiments has shown that catecholamine is a neurotransmitter containing catechol and amine groups. It can slow down the dynamic process of Aβ and αS fibrosis, inhibit the aggregation of Aβ and reduce aggregate toxicity [51].

In this study, the most important feature of FCF intervention in CD mice is the upregulation of metabolites beneficial to neuroprotection and anti-inflammation, especially actinonin, genipin, DHA-ee and enoxacin. On the contrary, after the supplement of FCF, the level of harmful metabolites such as diamino-5-formamidopyrimidine and 4-hydroxybutyric acid decreased. Lv et al. studied the neuroprotective effects of genipin on neuroinflammation and memory impairment in AD transgenic mice. The results showed that genipin could significantly inhibit the accumulation of RAGE-related signals, tumor necrosis factor-α (TNF- α), IL-1β and cerebral Aβ. Further studies have indicated that genipin could reduce the weakening effect of Aβ on the long-term potentiation of hippocampal neurons, increase the amplitude and frequency of miniature excitatory postsynaptic currents and increase synaptic plasticity of hippocampal neurons. In addition, oral administration of geniposide in APP/PS1 mice can improve the learning and memory ability of mice [52]. DHA-ee is ethyl ester DHA. DHA, as a fatty acid, can enhance memory and thinking ability, improve intelligence and so on. When the intake of DHA and related components is sufficient, the synaptic protein synthesis in the cerebral nerve increases and the transmission speed of neurons increases as well. Moreover, it was found that DHA could reduce the expression levels of IL-1 and IL-6 related to AD. Enoxacin is a cancer-specific growth inhibitor with strong activity against gram-positive and gram-negative bacteria. It plays a role by enhancing microRNA processing mediated by TARrna-binding protein 2(TRBP) [53]. Correlation analysis showed that genipin was positively correlated with *Bacteroides*, *Bacteroidetes* was positively correlated with DHA-ee and *Allobaculum* was positively correlated with enoxacin. 4,6-diamino-5-formamidopyrimidine is an oxidized DNA base, and oxidized nucleotides are biochemical markers of tumor aging and neurodegenerative diseases. Moreover, oxidative stress damage to DNA bases may lead to neuronal loss in AD [54]. It can be concluded that FCF intervention regulated intestinal microbiota to promote the release of beneficial metabolites in mice with circadian rhythm. We speculate that FCF and intestinal flora play a synergistic role in protecting the function of the intestine and hippocampus. Intestinal microorganisms can produce a lot of metabolites that affect rejection and the whole body, such as short-chain fatty acids that promote anti-inflammatory effects. However, the disturbance of intestinal flora caused by circadian rhythm disorder may cause intestinal damage and cognitive impairment [29,36]. Flavonoids have been proven to interact with intestinal flora. After ingestion, flavonoids regulate the diversity and structure of intestinal flora and the content of short-chain fatty acids in the host. On the other hand, the process of biotransformation mediated by intestinal flora also plays an important role in improving the bioavailability and activity expression of flavonoids [19]. In future research, it is necessary to focus on excavating the key signal pathways and specific intestinal flora of FCF to improve intestinal injury and brain function decline, and to explore the effects of intestinal flora related to circadian rhythm. For the dose-dependent relationship in which FCF plays a role, further repeated validation experiments are needed to determine its true dose–effect relationship.

## 5. Conclusions

In conclusion, FCF improved colonic epithelial lesions and submucosal vascular dilation in mice with circadian disturbance and reversed intestinal microflora structure abnormalities while improving the performance of rhythmic-disrupted mice on cognitive behavior tests. Mechanism research showed that FCF regulated metabolic pathways and related metabolites, upregulated the expression of related tight junction proteins in the mouse colon and regulated the levels of Aβ and inflammatory factors in the hippocampus. Further analysis found that these metabolites showed a certain correlation with intestinal flora and exerted a certain role in alleviating intestinal physiological damage and cognitive decline.

## Figures and Tables

**Table 1:** The contents of flavonoids in the FCF extract.

Compound	Content (mg/g)
rutin	169.77 ± 13.27
vitexin	143.52 ± 14.81
apigenin	119.98 ± 11.51
epicatechin	38.69 ± 1.63
luteolin	23.41 ± 0.85

**Table 2:** Effects of FCF on the biodiversity of gut microbiota in the circadian rhythm disorder mice model.

Sample	Chao1	Shannon	Simpson
CT-0	846.03 ± 32.95 ^a^	5.38 ± 0.54 ^b^	0.96 ± 0.03 ^c^
CT-4	879.61 ± 34.65 ^c^	8.04 ± 0.51 ^d^	0.99 ± 0.02 ^d^
CD-0	841.08 ± 43.05 ^a^	5.26 ± 0.54 ^b^	0.94 ± 0.07 ^c^
CD-4	846.06 ± 31.15 ^a^	4.34 ± 0.51 ^a^	0.85 ± 0.02 ^a^
FCF-0	840.26 ± 44.15 ^a^	5.28 ± 0.28 ^b^	0.91 ± 0.04 ^b^
FCF-4	868.56 ± 30.19 ^b^	7.12 ± 0.59 ^c^	0.92 ± 0.02 ^b^

Different letters in the same column indicate significant differences (*p* < 0.05) among different samples.

## Data Availability

The datasets generated for this study are available on request to the corresponding author.

## Supplementary Materials

The following supporting information can be downloaded at: https://www.mdpi.com/article/10.3390/foods12081682/s1, Figure S1: Representative elution profile of FCF by HPLC analysis. Figure S2: Effect of FCF on the food intake (A) and water intake (B) of the circadian rhythm disorder mice; Figure S3: Colon tissue injury score of each group; Figure S4: Principal component analysis of the gut metabolites among the three groups; Supporting Information.
