# Supplementary material for: Effects of Ficus pandurata Hance var. angustifolia Cheng Flavonoids on Intestinal Barrier and Cognitive Function by Regulating Intestinal Microbiota
Source: Foods. 2023 Apr 18;12(8):1682. doi: 10.3390/foods12081682 (PMC10137925; doi:10.3390/foods12081682)
Supplement: Supplementary file 1 [file foods-12-01682-s001.zip › foods-2235702-supplementary.pdf]

Lipid Profiles of Human Milk and Infant Formulas: A Comparative Lipidomics Study

Supplementary Materials

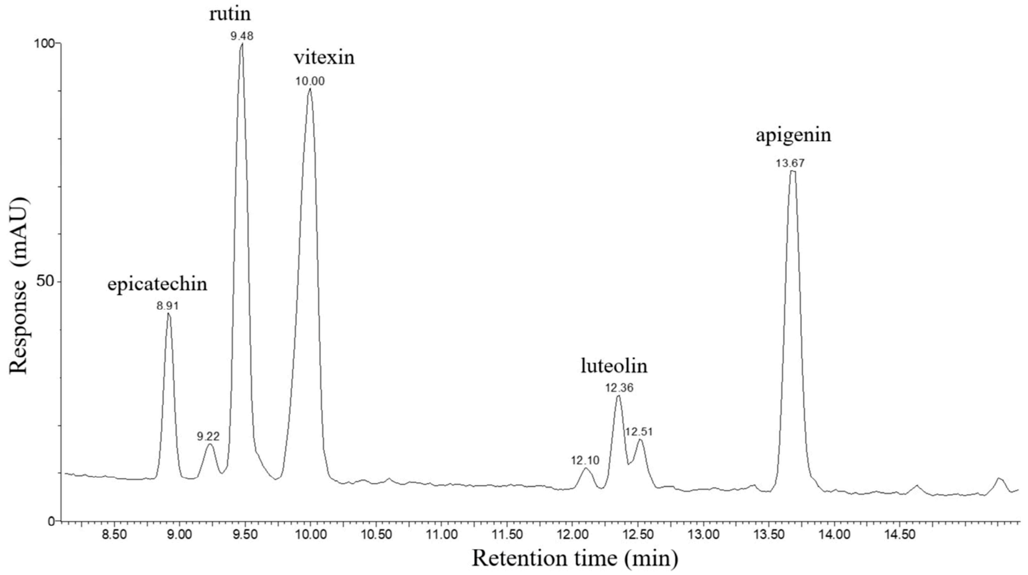

Figure S1. Representative elution profile of FCF by HPLC analysis.

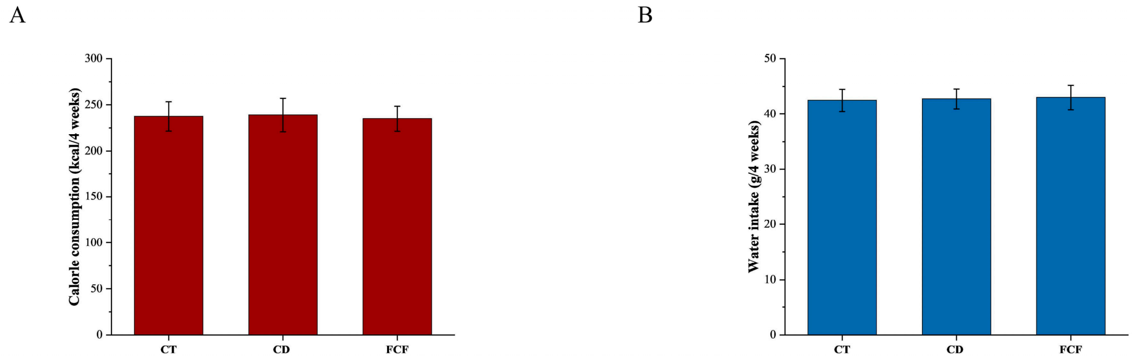

Figure S2. Effect of FCF on the food intake (A) and water intake (B) of the circadian rhythm disorder mice.

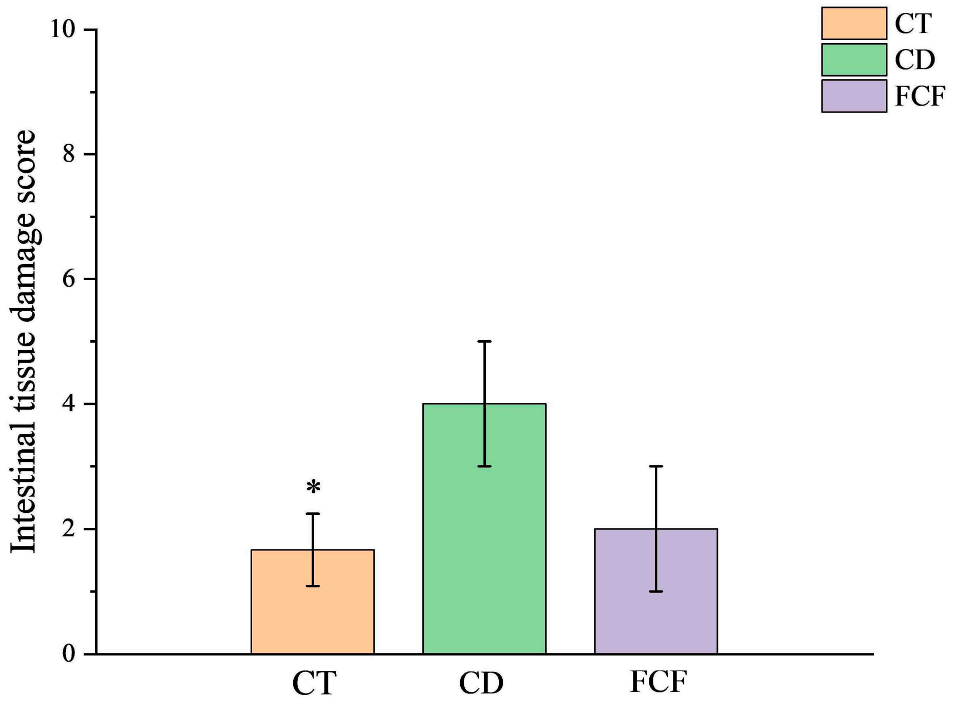

Figure S3. Colon tissue injury score of each group.

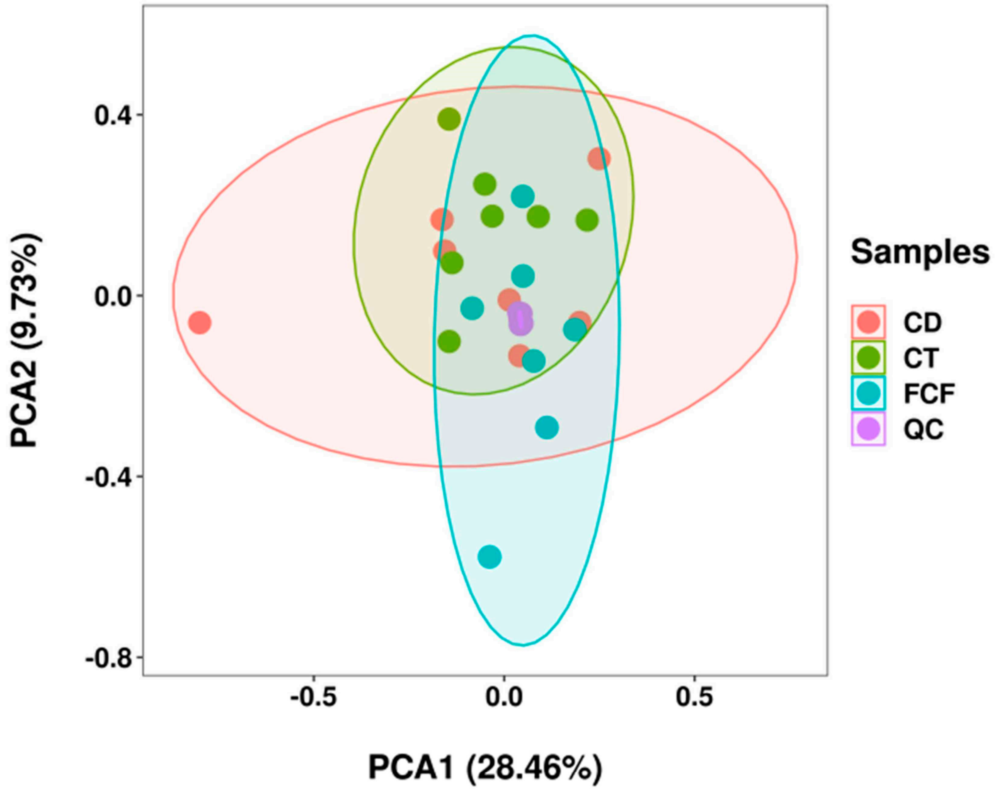

Figure S4. Principal component analysis of the gut metabolites among the three groups.

Supporting Information

1. Behavioral assessment

The Y-maze spontaneous alternation test

The Y-maze spontaneous alternation test is used to evaluate the short-term working memory ability of mice. Five mice in each group were randomly selected for the maze test. After 1 h darkness adaptation, all mice were placed in the center of the Y-maze device and explored freely in the Y maze for 8 min. The sequence and the total number of arms were recorded, and the correct spontaneous alternation rate was calculated. Correct rate of spontaneous alternation = [correct spontaneous alternation times / (total number of arm advance - 2 times)] × 100%.

The open-field test

At the beginning of the experiment, the mice were placed lightly in the central area and allowed to explore freely for 30 min. Their movement distance and times of entering the central area were recorded.

2. Histological examination

Put the sections into BioDewax and Clear Solution I for 15 min - BioDewax and Clear Solution II for 15 min - BioDewax and Clear Solution III for 15 min -absolute ethanol I for 5 min - absolute ethanol II for 5 min - 85% alcohol for 5 min - 75% alcohol for 5 min - rinse in distilled water. The slices were dyed with hematoxylin solution for 3-5 min, washed with tap water, differentiated with differentiation solution, washed with tap water, and rinsed with running water after returning to a blue solution. Sections were dehydrated with 85% and 95% gradient alcohol for 5 min each, dyed with eosin dye for 5 min, then dehydrated and sealed.

3. Analysis of intestinal microbiota

Using 1.2% agarose gel electrophoresis verification, for the qualified DNA, the V3-V4 variable region of each sample was amplified by primers and sequenced and read on the IlluminaMiSeq platform. QIIME (Quantitative Insights into Microbial Ecology) V1.2.8 was used to analyze the sequences. Vsearch (V2.3.4) clustered the sequences with more than 97% similarity into the same operational taxon (OTU). According to the OTU abundance of different species, the sequencing depth was measured by the Alpha diversity index. According to the representative sequences of each OUT and the comparison results of the 16S rRNA database (RDP and NT-16S), the species classification statistics of OTU were carried out, and species abundance tables at different classification levels were obtained.

4. Metabolite Extraction and LC–MS Analysis

All samples were analyzed by TripleTOF 5600 Plus high-resolution tandem mass spectrometer (SCIEX, Warrington, UK) for positive and negative ion patterns. Ultra performance liquid chromatography (UPLC) system (SCIEX, UK) was used for chromatographic separation. The reversed-phase separation was performed on an ACQUITYUPLC T3 column (100 mm * 2.1 mm, 1.8 um, Waters, UK). XCMS software was used to preprocess the collected LC-MS mass spectrometry data. The open-access databases KEGG and

HMDB were used to match the accurate molecular mass data (M/Z) with the threshold of 10 PPM data in the database, and the metabolites were annotated.
